# Mind the delay: duration of untreated psychosis is associated with short-term outcomes in first-episode schizophrenia

**DOI:** 10.3389/fpsyt.2026.1815016

**Published:** 2026-04-29

**Authors:** Dinghui Wang, Kaiguo Zhang, Zhi Xun Zhang, Qingqing Zhang, Min Liu, Yanhong Huang, Zhenqiang Xu

**Affiliations:** 1 Mental Health Center of Shantou University, Shantou University Medical College—Faculty of Medicine of University of Manitoba Joint Laboratory of Biological Psychiatry, Shantou, Guangdong, China; 2 Department of Preventive Medicine, Shantou University Medical College, Shantou, Guangdong, China; 3 Shenzhen Mental Health Centre, Shenzhen Kangning Hospital, Shenzhen, China

**Keywords:** Bayesian linear regression, duration of untreated psychosis, first-episode schizophrenia, propensity score matching, schizophrenia

## Abstract

**Background:**

Schizophrenia is a severe mental disorder associated with substantial disability. Although a growing body of evidence suggests that the duration of untreated psychosis (DUP) may influence prognosis, findings remains inconsistent.

**Methods:**

We retrospectively analyzed 99 first-episode schizophrenia patients hospitalized at Shantou University Mental Health Center between 2015 and 2019. DUP was categorized as short (≤26 weeks) or long (>26 weeks). Symptom improvement was assessed as changes in Brief Psychiatric Rating Scale (BPRS) score from admission to discharge. Propensity score matching was used to improve baseline comparability between DUP groups. A Bayesian linear regression model was then fitted to evaluate the adjusted association between DUP and BPRS improvement. Subgroup analyses were performed to assess whether the association varied across clinical characteristics.

**Results:**

In the unmatched cohort, patients with short DUP showed greater BPRS improvement than those with long DUP (mean difference 7.23 points, 95% CI 2.92 to 11.53). After matching, 22 pairs of patients were retained, and the association remained evident, with a larger improvement in the short DUP group (mean difference 11.50 points, 95% CI 5.92 to 17.08; *P < 0.001*). In the adjusted Bayesian model, long DUP remained associated with less symptom improvement (posterior median β = -3.93, 95% CrI -6.93 to -0.91, pd = 0.994), whereas higher baseline BPRS score was associated with greater improvement (posterior median β = 0.66, 95% CrI 0.55 to 0.77). Subgroup analyses suggested that the adverse association of long DUP was more pronounced among patients with higher baseline symptom severity (interaction *P = 0.014*).

**Conclusions:**

Shorter DUP was associated with greater symptom improvement in hospitalized patients with first-episode schizophrenia, which remained after matching and covariate adjustment and appeared stronger among patients with higher baseline symptom severity. These findings support DUP as a clinically useful prognostic indicator and reinforce the importance of earlier detection and timely intervention in first-episode schizophrenia.

## 1 Introduction

Schizophrenia is a chronic, severe psychiatric disorder that imposes a substantial global burden and remains a major therapeutic challenge (1). This disorder is often associated with impairments in cognition and functioning and may follow a heterogeneous clinical course, and is considered by clinicians and neuroscientists to be a progressive brain disease that can be associated with enduring symptoms and functional impairment in a subset of patients (2, 3). It affects approximately 20 million people worldwide and is among the top causes of disability globally, accounting for roughly 13.4 million years lived with disability (about 1.7% of total global YLDs) according to global disease estimates (4). In this context, duration of untreated psychosis (DUP), whose definition is the time from psychosis onset to initiation of adequate treatment, has emerged as a key indicator of treatment delay and a potentially modifiable factor in the course of illness (5–8). Early intervention paradigms propose a “critical period” in the early years of psychotic illness, during which prompt and sustained treatment may yield the greatest clinical benefit (9, 10).

Consistent with the early intervention framework, a substantial body of evidence links shorter DUP to better symptomatic and functional outcomes, whereas delayed treatment has been associated with more persistent symptoms, lower remission rates, and increased risk of adverse events. For example, a recent meta-analysis indicated that even brief delays (e.g., four vs. one week) are associated with significantly greater symptom severity, and that long DUP may halve the odds of achieving remission and double the risk of suicide attempts (11). Despite these findings, important uncertainties remain regarding the magnitude, specificity, and causal interpretability of the DUP–outcome association. First, the magnitude of this relationship is often modest, suggesting other clinical or sociodemographic factors may play a role. Some have argued that DUP may partly reflect underlying illness characteristics—such as insidious onset (12, 13), prominent negative symptoms (14), or barriers to care (15)—that themselves portend a worse prognosis. Moreover, the observational nature of most studies raises concerns about residual confounding due to baseline differences between patients with short and long DUP.

Another limitation of the existing literature is the lack of data from Asian settings (11). Most prior research has been conducted in high-income countries, despite known regional differences in help-seeking behavior, healthcare infrastructure, and treatment accessibility. Notably, DUP durations tend to be longer in Asian populations. A recent review reported an average DUP of nearly 49 weeks in Asia, compared to approximately 39 weeks in Europe (16). There are also significant differences within countries (17), for example, the average DUP in northern India is 18 weeks longer than that in southern India, almost twice the difference (18). These disparities underscore the need for studies in underrepresented regions, where cultural and systemic factors may shape both DUP and its clinical impact (19). The mechanisms by which DUP influences outcomes are still poorly understood. Few studies have formally examined potential mediating factors, such as early symptom stabilization, that may help explain the clinical benefits of prompt treatment.

The present study was designed to evaluate the association between DUP and short-term treatment outcomes in hospitalized patients with first-episode schizophrenia from South China. We hypothesized that shorter DUP would be associated with greater symptom improvement, and that this association would be more pronounced among patients with higher baseline symptom severity. To test these hypotheses, we used propensity score matching, adjusted Bayesian regression, and subgroup analyses. This study aims to clarify the prognostic role of DUP in early inpatient care and to inform strategies for earlier intervention.

## 2 Methods

### 2.1 Participants

This study included patients who were hospitalized at the Mental Health Center of Shantou University between January 2015 and March 2019 and were diagnosed with schizophrenia for the first time. Data collection included basic demographic information (e.g., sex, age, years of education), medical history, and lifestyle habits (e.g., smoking, alcohol consumption). The severity of psychiatric symptoms was evaluated using the BPRS at both admission and discharge. In addition, baseline metabolic indicators, including BMI, blood pressure, fasting glucose, blood lipids, and uric acid, were collected at admission. Detailed information on medication use was also recorded, including types of antipsychotics, treatment duration (as an indicator of intervention intensity), and whether polypharmacy was involved. Inclusion criteria were as follows: (1) A diagnosis of schizophrenia confirmed by at least two attending psychiatrists according to Diagnostic and Statistical Manual of Mental Disorders, Fifth Edition (DSM-5) criteria; (2) Age between 15 and 55 years, of Han ethnicity, with no gender restrictions; (3) First lifetime psychotic episode, with no prior use of antipsychotic medications before admission. Exclusion criteria included: (1) A history of severe chronic physical illnesses (e.g., hypertension, diabetes, thyroid disorders, cardiovascular or cerebrovascular diseases, hyperlipidemia, hepatic or renal dysfunction, infectious diseases), or long-term use of medications for physical conditions; (2) Incomplete medical records or missing assessment data; (3) History of or current use of psychoactive substances prior to admission (except for tobacco and alcohol, which were not considered exclusionary).

### 2.2 Interview and clinical assessment

The DUP was determined by a dual independent–extraction method: clinical data for each patient were independently abstracted by two researchers and entered into a predesigned data collection form. DUP was assessed by evaluating the onset timing and frequency of 16 common psychotic symptoms; any discrepancies or large differences between the two extractors prompted a joint re-evaluation until consensus was reached. The Schizophrenia Onset Symptom Scale (SOS) was used to quantify DUP, as it has demonstrated excellent inter-rater reliability (intraclass correlation coefficient [ICC] up to 0.97 for overall illness duration and kappa > 0.7 for most prodromal and psychotic symptom items) (20). Based on previous research, patients were classified into a “Short DUP” group (≤26 weeks) or “Long DUP” group (>26 weeks) (21, 22). First-episode schizophrenia patients were defined as individuals experiencing a first lifetime psychotic episode who met DSM-5 criteria for schizophrenia, with the diagnosis confirmed by at least two attending psychiatrists, and with no prior antipsychotic exposure before admission. Psychotic symptom severity was rated by attending psychiatrists or senior physicians using the Brief Psychiatric Rating Scale (BPRS), which is widely adopted in international collaborative studies. The BPRS comprises five symptom dimensions: (1) Anxiety-Depression; (2) Anergy (Lack of Energy); (3) Thought Disorder; (4) Activation; (5) Hostility-Suspicion. Each item is rated on a 7-point scale: 1 = Not present; 2 = Questionable or very mild; 3 = Mild; 4 = Moderate; 5 = Moderately severe; 6 = Severe; 7 = Extremely severe. Total scores range from 18 to 126, with higher scores indicating greater overall severity. Because the BPRS is an ordinal scale, it sensitively reflects treatment effects. The primary endpoint was the BPRS improvement score (admission minus discharge), calculated as admission BPRS minus discharge BPRS; larger positive values indicate greater symptom improvement.

### 2.3 Definition of key variables

Metabolic syndrome was defined according to the 2004 Chinese Diabetes Society (CDS) criteria. Specifically, participants were classified as having metabolic syndrome when three or more of the following were present: (1) BMI ≥25 kg/m²; (2) fasting plasma glucose ≥6.1 mmol/L, 2-h post-load glucose ≥7.8 mmol/L, or previously diagnosed diabetes; (3) blood pressure ≥140/90 mmHg or antihypertensive treatment; and (4) dyslipidemia, defined as triglycerides ≥1.7 mmol/L and/or HDL-C <0.9 mmol/L in men or <1.0 mmol/L in women. First-generation antipsychotic use was defined as receipt of at least one typical antipsychotic (e.g., sulpiride) during hospitalization. Second-generation antipsychotic use was defined as receipt of at least one atypical antipsychotic (e.g., risperidone, clozapine, olanzapine). Polypharmacy was defined as the concurrent use of two or more antipsychotic agents.

### 2.4 Statistical analysis

Descriptive analyses were performed in SPSS version 27.0. Data distributions were assessed for normality using formal tests and Q–Q plots. For normally distributed continuous variables, group comparisons were conducted using Welch’s t-test or one-way analysis of variance (ANOVA); for non-normal variables, the Wilcoxon rank-sum test or Kruskal–Wallis test was applied. Categorical variables were compared with the chi-square test, except where expected counts were < 5—in which case Fisher’s exact test was used.

Propensity score matching was implemented in R (version 4.2.2) using the MatchIt package (23) to calculate standardized mean differences (SMDs). The following baseline demographic and clinical covariates measured at admission were included in the propensity score model: gender, age, medical history, family history, smoking history, alcohol use history, presence of metabolic syndrome, years of education, body mass index (BMI), systolic blood pressure (SBP), diastolic blood pressure (DBP), fasting glucose, triglycerides (TG), high-density lipoprotein cholesterol (HDL-C), total cholesterol (TC), low-density lipoprotein cholesterol (LDL-C), and serum uric acid. ΔBPRS was then compared between groups in the matched sample using t-test, and mean differences with 95% confidence interval (CI) were reported; consistency between pre- and post-match estimates served as a sensitivity check. Statistically significant was considered any P value < 0.05. A Bayesian linear regression model was fitted in the full cohort to estimate the association between DUP category and BPRS improvement score in R (version 4.2.2), using the brms package (version 2.18.0) as an interface to Stan, adjusting for the above covariates. Posterior inference was based on Markov chain Monte Carlo (MCMC) sampling with 4 chains, 2,000 iterations per chain, and 1,000 warm-up iterations. Priors for all model parameters were specified as normal distributions centered at zero. Results are reported as posterior median regression coefficients (β) with 95% credible intervals (CrIs). The probability of direction (pd) was used to summarize evidence for the direction of the effect, with pd > 0.95 interpreted as considerable evidence of a directional association. Model convergence and sampling stability were assessed using the potential scale reduction factor (Rhat < 1.01) and effective sample size (ESS > 1000). Estimated marginal effects were visualized for variables of primary interest or with notable posterior associations to facilitate interpretability, while variables with negligible effects were omitted from visualization.

## 3 Result

### 3.1 Demographic and clinical characteristics of hospitalized first-episode schizophrenia patients

This study included 99 patients in total. Baseline characteristics indicated that the median age of patients was 28 years (interquartile range (IQR): 21–39), and the median duration of hospitalization was 32 days (IQR: 20–61). In terms of demographic features, males accounted for 59.6% (95% CI: 49%–69%), and the median duration of education was 9 years (IQR: 6–12). Regarding disease characteristics, the median duration of untreated psychosis (DUP) was 12 weeks (IQR: 2–144), with 42.4% (95% CI: 33%–53%) categorized as having a long DUP. Physiological parameter showed a median BMI of 19.50 (IQR: 17.60–21.80), mean systolic blood pressure of 121 ± 12 mmHg, and median fasting blood glucose of 5.01 mmol/L (IQR: 4.49–5.42). Lipid profiles revealed a median total cholesterol level of 4.64 mmol/L (IQR: 3.88–5.09). Clinically, the median baseline BPRS score was 43 points (IQR: 38–52), decreasing to 22 points (IQR: 19–28) at discharge, with a median improvement of 20 points (IQR: 16–26). In terms of medication, 82.8% (95% CI: 74%–89%) of patients used second-generation antipsychotics, and 38.4% (95% CI: 29%–49%) received polypharmacy. For comorbid conditions, metabolic syndrome was present in 3% (95% CI: 0.6%–8.6%) of patients,.

### 3.2 Differences in baseline demographic and clinical characteristics of short and long DUP first-episode schizophrenia patients

**Table 1** indicates that the Short DUP group consisted of 57 patients, while the Long DUP group included 42 patients. Significant differences in baseline characteristics were observed between the two groups: patients with a long DUP had a significantly longer hospital stay (median: 60 vs. 27 days, p < 0.001), older age (median: 29 vs. 24 years, *P = 0.046*), and a greater total duration of medication use (median: 61 vs. 29 days, p < 0.001) compared to patients with a short DUP. The Long DUP group also exhibited higher systolic blood pressure (126 ± 12 vs. 118 ± 12 mmHg, *P = 0.001*). Conversely, the Short DUP group demonstrated a larger improvement in BPRS scores (median: 22 vs. 19, *P = 0.008*). Additionally, categorical analyses showed that patients in the Long DUP group were significantly more likely to have a smoking history (26.2% vs. 1.8%, *p < 0.001*) and to use first-generation antipsychotics (31.0% vs. 14.0%, *P = 0.042*). No significant differences were observed between groups regarding gender distribution, metabolic parameters, or most treatment-related variables. These findings highlight distinct clinical features associated with DUP duration.

**Table 1:** Patient demographics and baseline characteristics.

Characteristic	Overall, N = 99^1^	DUP category		Statistic	P-value
		Short DUP, N = 57^1^	Long DUP, N = 42^1^		
Hospitalization days	32 (20, 61)	27 (15, 36)	60 (27, 90)	583.50	**<0.001^2^**
Age	28 (21, 39)	24 (18, 39)	29 (25, 39)	915.50	**0.046^2^**
DUP weeks	12 (2, 144)	4 (1, 8)	192 (96, 480)	0.00	**<0.001^2^**
Years of education	9.0 (6.0, 12.0)	9.0 (6.0, 10.0)	9.0 (6.0, 12.0)	1,082.50	0.406^2^
BMI	19.88 ± 2.91	19.95 ± 2.97	19.78 ± 2.86	0.30	0.768^3^
Systolic BP	121 ± 12	118 ± 12	126 ± 12	-3.37	**0.001^3^**
Diastolic BP	78 ± 9	77 ± 9	80 ± 10	-1.71	0.091^3^
Fasting glucose	5.07 ± 0.99	5.07 ± 1.15	5.08 ± 0.73	-0.07	0.941^3^
TG	0.81 (0.68, 1.07)	0.81 (0.66, 1.06)	0.81 (0.70, 1.06)	1,170.50	0.854^2^
HDL-C	1.41 ± 0.38	1.46 ± 0.33	1.35 ± 0.44	1.34	0.185^3^
Total Cholesterol	4.60 ± 0.98	4.57 ± 0.95	4.65 ± 1.02	-0.40	0.687^3^
LDL-C	2.72 ± 0.89	2.61 ± 0.90	2.88 ± 0.87	-1.52	0.132^3^
Uric acid	380 ± 105	383 ± 120	377 ± 83	0.26	0.793^3^
Total days on medication	40 (20, 71)	29 (13, 49)	61 (36, 123)	577.50	**<0.001^2^**
BPRS baseline	46 ± 14	48 ± 15	44 ± 11	1.68	0.096^3^
BPRS discharge	24 ± 8	23 ± 8	25 ± 8	-1.39	0.168^3^
BPRS improvement score	20 (16, 26)	22 (17, 30)	19 (13, 23)	1,572.50	**0.008^2^**
Gender				1.51	0.218^4^
Male	59 (59.6%)	31 (54.4%)	28 (66.7%)		
Female	40 (40.4%)	26 (45.6%)	14 (33.3%)		
Family history				0.02	0.892^4^
NO	70 (70.7%)	40 (70.2%)	30 (71.4%)		
YES	29 (29.3%)	17 (29.8%)	12 (28.6%)		
Smoking history				13.56	<0.001^4^
NO	87 (87.9%)	56 (98.2%)	31 (73.8%)		
YES	12 (12.1%)	1 (1.8%)	11 (26.2%)		
Alcohol use history					0.073^5^
NO	96 (97.0%)	57 (100.0%)	39 (92.9%)		
YES	3 (3.0%)	0 (0.0%)	3 (7.1%)		
Metabolic syndrome					0.073^5^
NO	96 (97.0%)	39 (92.9%)	57 (100.0%)		
YES	3 (3.0%)	3 (7.1%)	0 (0.0%)		
Used first generation antipsychotic				4.14	0.042^4^
NO	78 (78.8%)	49 (86.0%)	29 (69.0%)		
YES	21 (21.2%)	8 (14.0%)	13 (31.0%)		
Used second generation antipsychotic				2.26	0.133^4^
NO	17 (17.2%)	7 (12.3%)	10 (23.8%)		
YES	82 (82.8%)	50 (87.7%)	32 (76.2%)		
Polypharmacy				0.62	0.432^4^
NO	61 (61.6%)	37 (64.9%)	24 (57.1%)		
YES	38 (38.4%)	20 (35.1%)	18 (42.9%)		

^1^Median (IQR); Mean ± SD; n (%).

^2^Wilcoxon rank sum test.

^3^Welch Two Sample t-test.

^4^Pearson’s Chi-squared test.

^5^Fisher’s exact test.

Bold values indicate statistically significant differences between the Short DUP and Long DUP groups (P < 0.05).

### 3.3 Baseline covariate balance achieved through propensity score matching

A total of 99 first-episode hospitalized schizophrenia patients (57 Short DUP, 42 Long DUP) were included in the analysis. To minimize confounding by baseline characteristics, we performed 1:1 propensity score matching using a genetic matching algorithm with a caliper width of 0.2 on the logit of the propensity score. The matching procedure included the following baseline covariates: gender, age, family history, smoking history, alcohol use history, years of education, body mass index (BMI), systolic and diastolic blood pressure, fasting glucose, triglycerides, high-density lipoprotein cholesterol (HDL-C), total cholesterol, low-density lipoprotein cholesterol (LDL-C), and uric acid.

After matching, 22 Short DUP patients were successfully paired with 22 Long DUP patients (n = 44). Covariate balance between the two groups was assessed using standardized mean differences (SMDs), with SMDs < 0.10 considered indicative of negligible imbalance. As shown in **Table 2**, baseline imbalance was substantially reduced after matching. The Love plot before and after matching is presented in **Supplementary Figure S1**.

**Table 2:** Baseline covariate balance between short and long DUP groups before and after propensity score matching.

Variables	Level	Before matching			After matching		
		Short DUP	Long DUP	SMD^△^	Short DUP	Long DUP	SMD^△^
n		57	42		22	22	
Systolic BP (mean (SD))		117.61 (11.88)	125.67 (11.64)	0.692	123.32 (11.34)	122.64 (11.78)	-0.059
Smoking history (%)	NO	56 (98.2)	31 (73.8)	-0.556	21 (95.5)	20 (90.9)	-0.103
	YES	1 (1.8)	11 (26.2)	0.556	1 (4.5)	2 (9.1)	0.103
Diastolic BP (mean (SD))		76.88 (8.71)	80.12 (9.76)	0.332	80.55 (10.24)	79.68 (11.19)	-0.089
LDL-C (mean (SD))		2.61 (0.90)	2.88 (0.87)	0.314	2.71 (0.85)	2.74 (0.56)	0.033
Age (mean (SD))		29.44 (13.34)	33.12 (12.35)	0.298	28.23 (11.46)	29.05 (11.48)	0.066
Alcohol use history (%)	NO	57 (100.0)	39 (92.9)	-0.277	22 (100.0)	22 (100.0)	0.000
	YES	0 (0.0)	3 (7.1)	0.277	0 (0.0)	0 (0.0)	0.000
Gender (%)	Female	26 (45.6)	14 (33.3)	-0.261	12 (54.5)	11 (50.0)	-0.096
	Male	31 (54.4)	28 (66.7)	0.261	10 (45.5)	11 (50.0)	0.096
HDL-C (mean (SD))		1.46 (0.33)	1.35 (0.44)	-0.245	1.30 (0.18)	1.33 (0.24)	0.064
Years of education (mean (SD))		8.26 (3.89)	9.00 (3.78)	0.195	8.45 (3.28)	8.50 (4.24)	0.012
Total Cholesterol (mean (SD))		4.57 (0.95)	4.65 (1.02)	0.080	4.46 (0.92)	4.50 (0.62)	0.041
Uric acid (mean (SD))		382.70 (120.07)	377.33 (82.66)	-0.065	355.82 (96.70)	375.50 (84.64)	0.238
BMI (mean (SD))		19.95 (2.97)	19.78 (2.86)	-0.061	20.03 (3.10)	19.86 (3.23)	-0.060
TG (mean (SD))		0.96 (0.57)	0.93 (0.40)	-0.056	0.97 (0.69)	0.95 (0.40)	-0.039
Family history (%)	NO	40 (70.2)	30 (71.4)	0.028	17 (77.3)	16 (72.7)	-0.101
	YES	17 (29.8)	12 (28.6)	-0.028	5 (22.7)	6 (27.3)	0.101
Fasting glucose (mean (SD))		5.07 (1.15)	5.08 (0.73)	0.019	5.15 (0.84)	5.11 (0.80)	-0.061

^△^Standardized Mean Difference

### 3.4 Association between DUP category and symptom improvement before and after matching

Both unmatched and matched analyses demonstrated that a shorter DUP was associated with significantly greater symptom improvement, as reflected by a larger BPRS improvement score, after treatment. In the unmatched cohort (n = 99), the Short DUP group had a higher mean BPRS improvement score than the Long DUP group (25.4 vs. 18.1), with a mean difference of 7.23 points (95% CI: 2.92 to 11.53, *P = 0.001*) (**Supplementary Tables S2**, **3**). In the matched cohort (n = 44), the effect size was even larger. The Short DUP group showed a mean BPRS improvement score of 29.2 ± 12.79, whereas the Long DUP group showed 17.7 ± 7.56, yielding a mean paired difference of 11.50 points (95% CI: 5.92 to 17.08, *P < 0.001*) (**Tables 3**, **4**). Despite the reduced sample size after matching, the consistency in direction and magnitude supported the robustness of the main association.

**Table 3:** BPRS improvement score in the matched cohort.

Treatment	BPRS improvement score	
	N	Mean (SD)
Short DUP	22	29.2 (12.79)
Long DUP	22	17.7 (7.56)

SD, Standard Deviation.

**Table 4:** Between-group comparison of BPRS improvement scores in the matched cohort.

Pairwise comparison	Mean difference (95% CI) ^a^	P-value
Short DUP - Long DUP	11.50 (5.92, 17.08)	<0.001

^a^Paired t-test comparison of BPRS improvement scores in the matched cohort.

^a^ *Paired t-test comparison of BPRS improvement scores in the matched cohort.*

### 3.5 Bayesian linear modeling of factors associated with BPRS improvement

In the full cohort, a Bayesian linear regression model was fitted to examine the association between DUP category and BPRS improvement score while adjusting for baseline BPRS score, age, gender, years of education, BMI, uric acid, smoking history, and family history. The model showed good convergence and sampling stability, with Rhat values close to 1.00 and effective sample sizes greater than 3600, and demonstrated acceptable explanatory performance (R² = 0.67, adjusted R² = 0.62).

**Figure 1:**
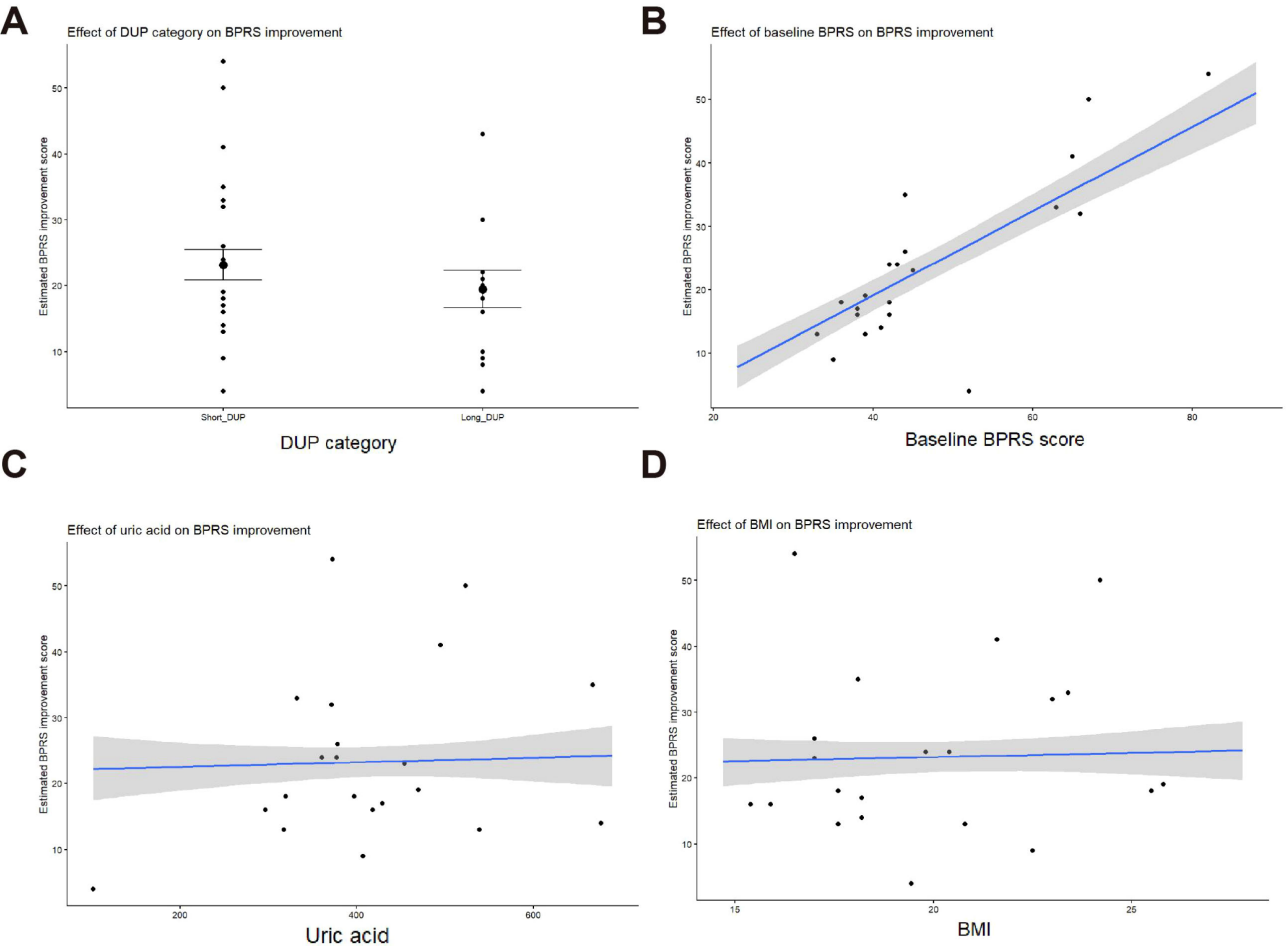
Marginal effects of key predictors on BPRS improvement score in first-episode hospitalized schizophrenia patients. **(A)** Effect of DUP category, **(B)** effect of baseline symptom severity, **(C)** effect of uric acid, and **(D)** effect of BMI on the BPRS improvement score in first-episode hospitalized schizophrenia patients.

Results (**Table 5**) revealed that, after adjustment, long DUP remained associated with a lower BPRS improvement score compared with Short DUP (posterior median β = -3.93, 95% CrI -6.93 to -0.91, pd = 0.994), indicating less symptom improvement in the Long DUP group. Baseline BPRS scores was positively associated with BPRS improvement (posterior median β = 0.66, 95% CrI 0.55 to 0.77, pd = 1.000), suggesting that patients with more severe initial psychiatric symptoms show greater improvement following treatment. The posterior estimates for age, gender, years of education, BMI, uric acid, family history and smoking history had 95% CrIs spanning zero, indicating limited evidence for robust independent associations with BPRS improvement in this model. To further elucidate these relationships, estimated marginal effects derived from the fully adjusted Bayesian model were visualized (**Figures 1A–D**). The results demonstrated that patients with Long DUP consistently showed lower predicted BPRS improvement compared to those with Short DUP, after controlling for covariates. Baseline BPRS score exhibited a strong positive relationship with symptom improvement, indicating that patients with more severe initial symptoms experienced greater reductions during hospitalization. In contrast, BMI and uric acid showed only modest associations with the outcome, with CrIs overlapping zero, suggesting limited independent effects.

**Table 5:** Bayesian linear model results for predictors of BPRS improvement score in first-episode hospitalized schizophrenia patients.

Parameter	Median (β)	2.5% CrI	97.5% CrI	pd	Rhat	ESS
(Intercept)	-11.248	-25.14075913	2.66639124	0.94825	0.9993056	4,346.203
DUP category	-3.932	-6.92704155	-0.90976599	0.99400	0.9999906	3,655.303
BPRS baseline	0.664	0.55491203	0.77387433	1.00000	0.9995571	4,547.783
Age	-0.0036	-0.12990483	0.12805747	0.51850	1.0004586	4,027.720
Gender	0.066	-3.07023393	3.31558987	0.51425	0.9993557	3,660.651
Years of education	0.005	-0.38915962	0.39623032	0.51250	0.9998492	4,375.445
BMI	0.134	-0.39494199	0.67999644	0.69625	1.0000635	4,108.230
Uric acid	0.003	-0.01011939	0.01754623	0.68550	0.9997913	3,901.076
Smoking history	-1.113	-5.75678252	3.35807921	0.68450	0.9999792	4,111.672
Family history	2.572	-0.32834913	5.64416867	0.96025	0.9997531	4,363.069

Results are based on a Bayesian linear regression model via MCMC sampling (4 chains, 2000 iterations each, 1000 warm-up). Median indicates the posterior median regression coefficient (effect size). Posterior medians and 95% CrIs are reported. pd represents the probability of direction, values > 0.95 are interpreted as considerable evidence of a directional effect. Rhat values < 1.01 and effective sample sizes (ESS) >1000 indicate good convergence and sampling stability. Priors for all parameters were specified as normal distributions centered at zero with parameter-specific scales. Model fit indices: expected log predictive density (ELPD) = –338.32; leave-one-out information criterion (LOOIC) = 676.63; widely applicable information criterion (WAIC) = 676.47; R² = 0.670; adjusted R² = 0.616; Residual SD (σ) = 6.91.

### 3.6 Subgroup analysis of the association between DUP and symptom improvement across clinical characteristics

**Figure 2:**
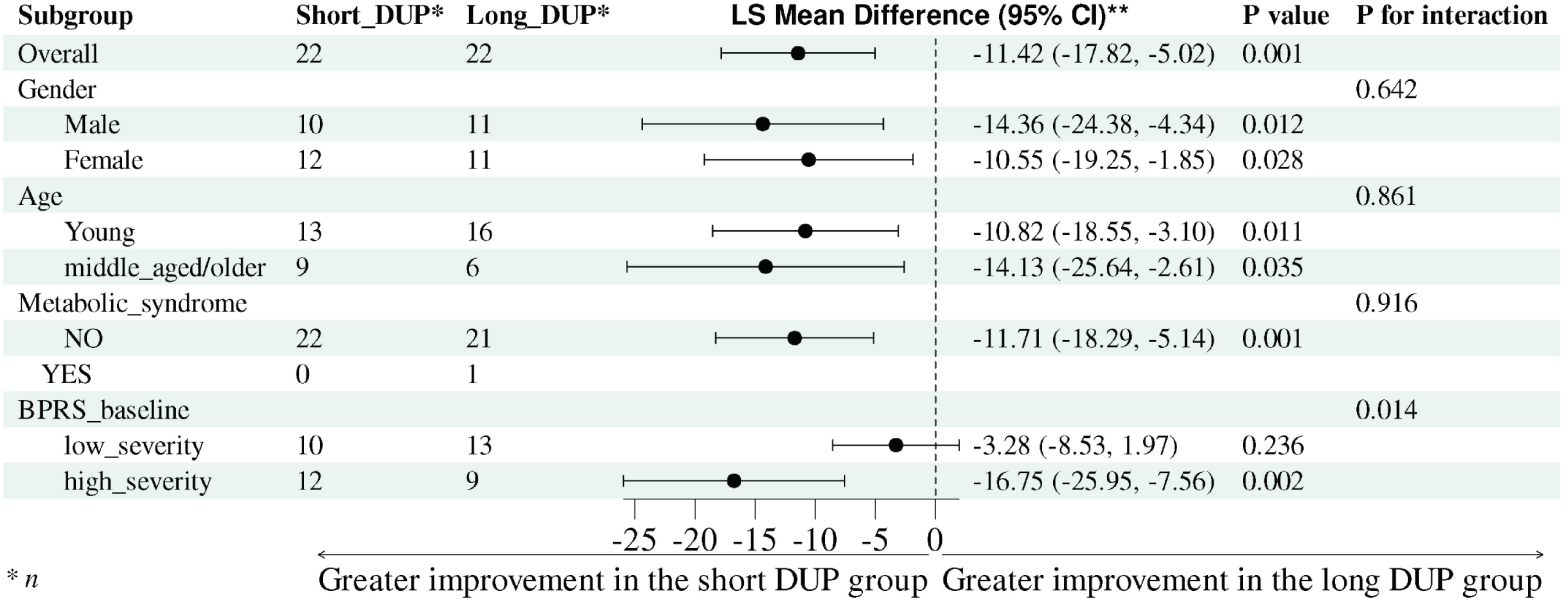
Forest plot of subgroup analyses of BPRS improvement score for short versus long DUP in hospitalized patients with first-episode schizophrenia (adjusted for BMI and uric acid). Each point represents the adjusted mean difference in BPRS improvement score (Short DUP minus Long DUP), and the horizontal lines indicate the 95% confidence intervals (CIs). “P value” denotes the within-subgroup test for the Short vs. Long DUP effect, and “P for interaction” denotes the interaction test for the DUP category × subgroup term in the full-sample model. * n, number of participants in each subgroup. ** LS mean difference (95% CI), adjusted least-squares mean difference between the Short DUP and Long DUP groups, with corresponding 95% confidence interval.

For subgroup analyses, we dichotomized age at 30 years (“young” < 30 vs. “middle-aged/older” ≥ 30) and baseline BPRS at 45 points (“low” vs. “high” severity). We then fitted separate models for each subgroup—gender, age group, metabolic syndrome status, and baseline severity—retaining DUP category as the key predictor and adjusting for BMI and uric acid. Overall, the Short DUP group showed significantly greater BPRS improvement than the Long DUP group, with an adjusted mean difference of 11.42 points in favor of the Short DUP group (95% CI: -17.82 to -5.02, *P = 0.001*). **Figure 2** show that this association was consistent across gender and age strata, with no evidence of interaction by gender (P for interaction = 0.642) or age group (P for interaction = 0.861). Significant associations were observed in both males (adjusted mean difference= -14.36, 95% CI: -24.38 to -4.34, *P = 0.012*) and females (adjusted mean difference= -10.55, 95% CI: -19.25 to -1.85, *P = 0.028*), as well as in both younger patients (95% CI= -10.82, 95% CI: -18.55 to -3.10, *P = 0.011*) and middle-aged/older patients (95% CI= -14.13, 95% CI: -25.64 to -2.61, *P = 0.035*). Metabolic syndrome did not appear to modify the association (P for interaction = 0.916); however, this subgroup analysis was not informative because only one matched patient had metabolic syndrome, and that patient was in the Long DUP group. By contrast, baseline BPRS severity significantly modified the association between DUP and symptom improvement (P for interaction = 0.014). The association was substantially stronger in the high-severity subgroup (95% CI= -16.75, 95% CI: -25.95 to -7.56, *P = 0.002*), whereas the effect in the low-severity subgroup was smaller and not statistically significant (95% CI= -3.28, 95% CI: -8.53 to 1.97, *P = 0.236*). These findings may suggest that longer DUP was associated with less symptom improvement during hospitalization across most clinical strata, with a particularly pronounced adverse association among patients presenting with higher baseline symptom severity.

## 4 Discussion

In this retrospective study of hospitalized patients with first-episode schizophrenia, shorter DUP was associated with greater short-term symptom improvement during hospitalization, and this align with previous studies (24, 25). While most literature supports the notion that longer DUP predicts poorer outcomes (11, 26, 27), some studies question the universality of this association (26, 28). At the same time, DUP is closely intertwined with other adverse clinical features, including help-seeking delay, symptom profile, and pathways to care, which may also influence treatment response (22, 26, 29–31). For that reason, DUP in the present study is best interpreted as a clinically relevant prognostic marker rather than proof of a direct causal effect. In our study, propensity score matching partially controlled for baseline differences between short and long DUP groups, suggesting that the association persisted after adjustment for measured baseline characteristics. Furthermore, no consensus currently exists regarding the threshold for defining a “long DUP,” with studies variably setting thresholds from 1 month to 1 year (7, 32).

The subgroup findings add an important layer to this interpretation. We found that the association between longer DUP and poorer symptom improvement was stronger in patients with higher baseline BPRS severity, whereas the corresponding association was smaller and not statistically clear in the lower-severity subgroup. Clinically, this suggests that treatment delay may be especially consequential in patients who already present with a high symptom burden at admission. DUP and baseline symptom severity may therefore be considered together when identifying patients at higher risk of limited short-term inpatient improvement.

The mechanisms underlying poorer treatment outcomes in prolonged DUP remain unclear but likely involve both clinical and psychosocial impairments. The “neurotoxicity hypothesis” suggests prolonged untreated psychosis may have toxic effects on the brain (33). First proposed by Wyatt et al. in 1991 (34), this hypothesis posits that persistent psychotic symptoms adversely affect brain tissue through excessive neuronal excitability, stress hormone release, and oxidative stress, potentially impairing response to antipsychotic treatment (33). However, evidence for a direct neurobiological effect of DUP remains mixed, and not all neuroimaging or neuropsychological studies support such an interpretation (35–38). These inconsistencies may reflect differences in case mix, follow-up duration, and outcome measurement. While our study did not directly measure brain structural or cognitive alterations, clinically it can be speculated that prolonged untreated periods may induce adaptive changes, attenuating subsequent treatment responses. Our findings fit more comfortably within the early-intervention and critical-period framework in psychosis (39). The initial five years following schizophrenia onset are considered pivotal for influencing the long-term disease trajectory (24, 40, 41), and we proposes that the period shortly after illness onset may be especially important for shaping subsequent trajectory, making treatment delay clinically meaningful even when causality cannot be established directly. Conversely, treatment delays during this critical period may lead to cumulative symptom burden, functional decline, and loss of social support—functional losses that may be difficult to fully reverse even with later treatment. Several clinical implications follow from these findings. First, DUP should be documented at first contact as an actionable clinical time marker rather than a descriptive historical detail. Second, patients with longer DUP may warrant closer monitoring of early inpatient response, because a smaller short-term reduction in BPRS may be more likely in this group. Third, the stronger association observed in patients with higher baseline BPRS scores suggests that DUP and baseline severity may be considered together when identifying patients at higher risk of limited short-term improvement. In our data, the association persisted after propensity score matching and covariate adjustment, which supports the clinical usefulness of DUP as a prognostic indicator during hospitalization. Clinically, this means that patients entering care after a prolonged untreated period may require more cautious expectations for short-term inpatient symptom improvement. At the service level, these findings support earlier recognition of psychotic symptoms by families and front-line providers, faster referral to psychiatric assessment, and treatment pathways that shorten avoidable delays before formal care begins.

This study’s strengths include focusing on first-episode schizophrenia patients, thus minimizing confounding factors such as multiple episodes, chronic illness, and prolonged medication exposure. We also combined baseline comparison, propensity score matching, adjusted Bayesian modeling, and subgroup analyses, which provided convergent evidence across analytic approaches. However, our study has limitations, including a relatively small sample size (N = 99), limiting detection of subtle effects and long-term outcomes. The retrospective observational design also cannot rule out unmeasured confounding, including differences in pathways to care, family support, insight, or treatment engagement. In addition, the analysis was restricted to hospitalized patients and focused on short-term symptom improvement measured by the BPRS, rather than cognition, daily functioning, or social recovery. Although symptom improvement is a primary outcome measure, comprehensive long-term prognosis assessments should incorporate these broader domains. Future studies may test whether the short-term disadvantage associated with longer DUP persists across longer follow-up and broader outcome domains, including cognition, functioning, relapse, and community reintegration. Larger prospective cohorts with extended follow-ups should verify whether short-term inpatient advantage associated with shorter DUP translates into better long-term clinical and functional outcomes. This may also be relevant to peripheral metabolic or oxidative biomarkers. For example, a recent systematic review in first-episode psychosis reported a tendency toward lower uric acid levels, although the evidence appeared to vary across populations (42). Additionally, employing high-resolution neuroimaging and dynamic peripheral biomarker monitoring may clarify whether extended DUP induces neurotoxicity or chronic stress responses. Prospective characterization of help-seeking pathways, family support, and barriers to care may also clarify whether DUP mainly reflects delayed treatment itself or a broader pattern of social and service disadvantage. This may be particularly relevant in Chinese community settings, where recent work has highlighted the roles of discrimination experiences and other modifiable contextual factors in schizophrenia-related adverse outcomes (43). Research on early-detection programs, streamlined referral systems, and continuity-of-care models may be clinically valuable, because reducing DUP is one of the few potentially modifiable targets highlighted by these findings.

## 5 Conclusions

In summary, this study suggests that a shorter DUP is associated with greater symptomatic improvement in first-episode hospitalized schizophrenia patients, particularly among those with more severe baseline symptoms. These findings support DUP as a clinically useful prognostic indicator during early inpatient care and suggest that DUP and baseline symptom severity may be considered together when identifying patients at higher risk of limited short-term improvement. From a clinical and service perspective, the results support earlier recognition of psychotic symptoms, faster psychiatric referral, and treatment pathways that reduce avoidable delays before formal care begins. Future prospective studies should examine long-term clinical and functional outcomes, neurobiological correlates, and sociocultural or health-system factors that contribute to treatment delay.

## Data Availability

The raw data supporting the conclusions of this article will be made available by the authors, without undue reservation.
