# Supplementary material for: Mind the delay: duration of untreated psychosis is associated with short-term outcomes in first-episode schizophrenia
Source: Front Psychiatry. 2026 Apr 29;17:1815016. doi: 10.3389/fpsyt.2026.1815016 (PMC13167566; doi:10.3389/fpsyt.2026.1815016)
Supplement: Supplementary file 1 [file DataSheet1.pdf]

Supplementary Material

1 Supplementary Figures

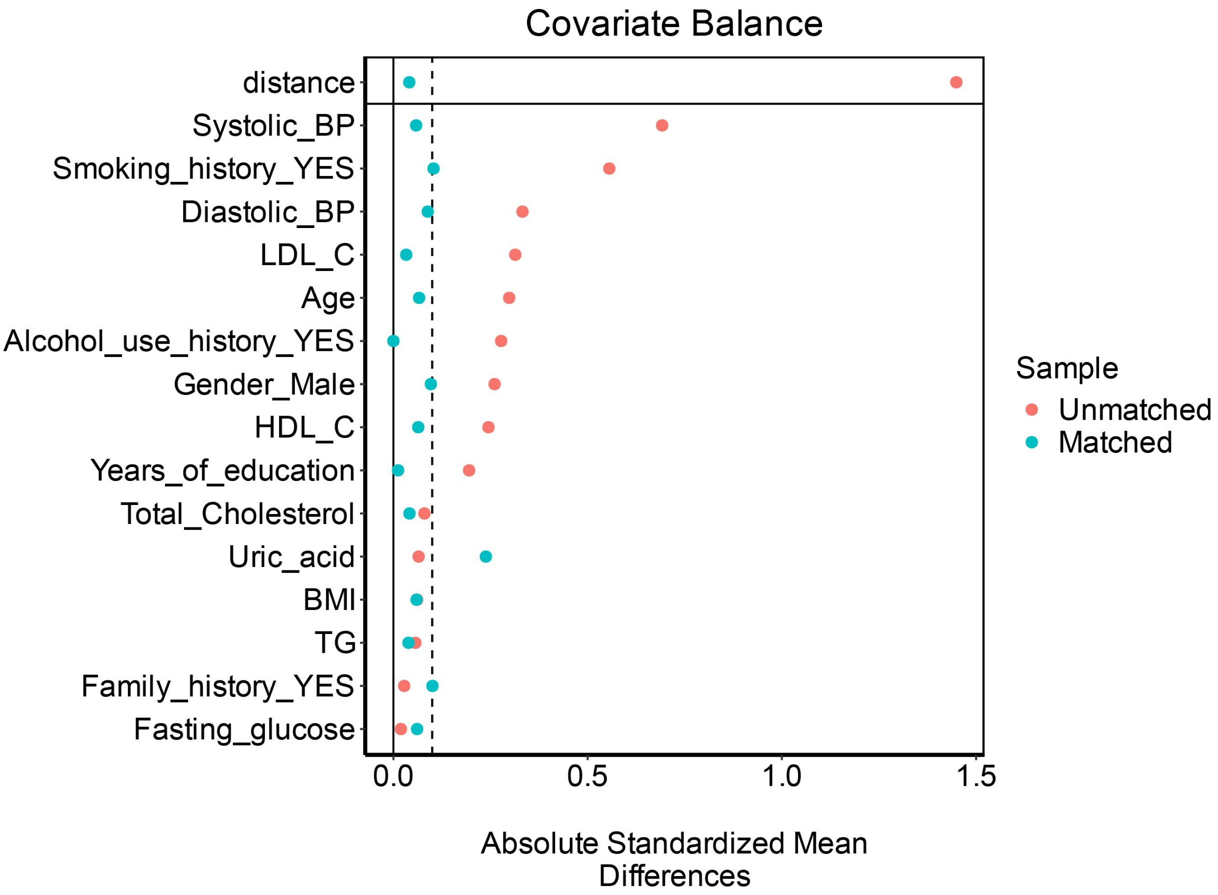

Figure S1. Love plot before and after matching

Table S1. Patient demographics and baseline characteristics

Characteristics	Unmatched			Matched		
	Short DUP, N = 57[1]	Long DUP, N = 42[1]	p-value[2]	Short DUP, N = 22[1]	Long DUP, N = 22[1]	p-value[3]
Gender			0.218			0.763
Male	31 (54%)	28 (67%)		12 (55%)	11 (50%)	
Female	26 (46%)	14 (33%)		10 (45%)	11 (50%)	
Age			0.046			0.814
Median (IQR)	24 (18, 39)	29 (25, 39)		28 ± 11	29 ± 11	
Family history			0.892			0.728
NO	40 (70%)	30 (71%)		17 (77%)	16 (73%)	
YES	17 (30%)	12 (29%)		5 (23%)	6 (27%)	
Smoking history			<0.001			>0.999
NO	56 (98%)	31 (74%)		21 (95%)	20 (91%)	
YES	1 (2%)	11 (26%)		1 (5%)	2 (9%)	
Alcohol use history			0.073			>0.999
NO	57 (100%)	39 (93%)		22 (100%)	22 (100%)	

Characteristics	Unmatched			Matched		
	Short DUP, N = 57[1]	Long DUP, N = 42[1]	p-value[2]	Short DUP, N = 22[1]	Long DUP, N = 22[1]	p-value[3]
YES	0 (0%)	3 (7%)		0 (0%)	0 (0%)	
Years of education			0.347			0.968
Mean ± SD	8.3 ± 3.9	9.0 ± 3.8		8.5 ± 3.3	8.5 ± 4.2	
BMI			0.768			0.859
Mean ± SD	19.95 ± 2.97	19.78 ± 2.86		20.0 ± 3.1	19.9 ± 3.2	
Systolic BP			0.001			0.846
Mean ± SD	118 ± 12	126 ± 12		123 ± 11	123 ± 12	
Diastolic BP			0.091			0.791
Mean ± SD	77 ± 9	80 ± 10		81 ± 10	80 ± 11	
Fasting glucose			0.941			0.858
Mean ± SD	5.07 ± 1.15	5.08 ± 0.73		5.15 ± 0.84	5.11 ± 0.80	
TG			0.819			0.928
Mean ± SD	0.96 ± 0.57	0.93 ± 0.40		0.97 ± 0.69	0.95 ± 0.40	
HDL-C			0.185			0.669
Mean ± SD	1.46 ± 0.33	1.35 ± 0.44		1.30 ± 0.18	1.33 ± 0.24	
Total Cholesterol			0.687			0.859
Mean ± SD	4.57 ± 0.95	4.65 ± 1.02		4.46 ± 0.92	4.50 ± 0.62	
LDL-C			0.132			0.896
Mean ± SD	2.61 ± 0.90	2.88 ± 0.87		2.71 ± 0.85	2.74 ± 0.56	
Uric acid			0.793			0.477
Mean ± SD	383 ± 120	377 ± 83		356 ± 97	376 ± 85	

[1]n (%); [2]Welch Two Sample t-test; Pearson's Chi-squared test; Fisher's exact test; [3]Welch Two Sample t-test; Fisher's exact test; Pearson's Chi-squared test

Table S2. Description for unmatched data showing mean change in BPRS scores for patients with short versus long DUP.

		BPRS score change
Treatment	N	Mean (SD)
Short DUP	57	25.4 (12.45)
Long DUP	42	18.1 (9.15)

Summary of BPRS score changes [mean (SD)] among patients with short versus long DUP, SD = Standard Deviation

Table S3. Independent-samples t-test comparison of BPRS improvement scores between Short and Long DUP groups in the unmatched cohort

Pairwise Comparison	Mean Difference (95% CI) [a]	p-Value
Short DUP - Long DUP	7.23 (2.92, 11.53)	0.001

[a] Based on Welch's two-sample t-test. CI = confidence interval
